# Extensive and Intimate Association of the Cytoskeleton with Forming Silica in Diatoms: Control over Patterning on the Meso- and Micro-Scale

**DOI:** 10.1371/journal.pone.0014300

**Published:** 2010-12-10

**Authors:** Benoit Tesson, Mark Hildebrand

**Affiliations:** Marine Biology Research Division, Scripps Institution of Oceanography, University of California San Diego, La Jolla, California, United States of America; Clarkson University, United States of America

## Abstract

**Background:**

The diatom cell wall, called the frustule, is predominantly made out of silica, in many cases with highly ordered nano- and micro-scale features. Frustules are built intracellularly inside a special compartment, the silica deposition vesicle, or SDV. Molecules such as proteins (silaffins and silacidins) and long chain polyamines have been isolated from the silica and shown to be involved in the control of the silica polymerization. However, we are still unable to explain or reproduce *in vitro* the complexity of structures formed by diatoms.

**Methods/Principal Finding:**

In this study, using fluorescence microscopy, scanning electron microscopy, and atomic force microscopy, we were able to compare and correlate microtubules and microfilaments with silica structure formed in diversely structured diatom species. The high degree of correlation between silica structure and actin indicates that actin is a major element in the control of the silica morphogenesis at the meso and microscale. Microtubules appear to be involved in the spatial positioning on the mesoscale and strengthening of the SDV.

**Conclusions/Significance:**

These results reveal the importance of top down control over positioning of and within the SDV during diatom wall formation and open a new perspective for the study of the mechanism of frustule patterning as well as for the understanding of the control of membrane dynamics by the cytoskeleton.

## Introduction

Diatoms are unicellular algae that make cell walls out of silica which is structured on the nano- to micro-scale in an enormous variety of shapes. The number of diatom species, each with a distinct shape, is estimated in the hundreds-of-thousands [1]. Diatoms range in overall size from two to several hundreds of microns, with detailed and intermediate features ranging from the nanometer to micron scale. Because of their ability to reproducibly form complex three dimensional structures with controlled features at multiple length scales, diatoms are an exceptional model for the study of silica biomineralization and the development of biomimetic approaches for nanoscale materials synthesis.

The diatom cell wall is called the frustule, which is arranged in two parts like a petri dish with an upper and lower overlapping half called the epi- and hypo-theca, respectively. Each theca consists of a valve, which is the distinctive structure characteristic of a given species and which caps the theca, and a series of overlapping girdle bands, which are most commonly thin silica bands that encircle the sides of the cell and provide the overlap between the two thecae. There are two general structural classes of diatoms, the centrics, which have radially symmetric valves, and the pennates, which are bilaterally symmetrical [2]. A subclass of the pennate diatoms has an elongated slit in the valve called the raphe. Adhesive mucilage which sticks to the surface the diatom is on is secreted through the raphe, and the mucilage interfaces with an intracellular actin/myosin motor protein system to enable gliding movement on surfaces [3]; [4].

Patterning and formation of the mineralized structures of the diatom frustule are carried out by organic compounds and organelles in the cell. Silica structures are formed in a specific compartment called the Silica Deposition Vesicle (SDV -[5]; [6]). After formation is complete, then the entire structure is exocytosed to contribute to the new wall. Silicon is transported into the cell in soluble form as silicic acid [7], which is eventually condensed inside the SDV to form solid silica. Silica structure in diatoms has been divided in three different scales [8]; [9]. The nanoscale represents the initial silica polymerization events, generating structures with up to a few tens-of-nanometers features. Characterization of organic components tightly associated with diatom silica has identified three classes of molecule that are likely to be the major players in nanoscale structure formation. These include highly modified (poly)peptides called silaffins [10]–[12], unique long chain polyamines (LCPAs -[13]) that are not found elsewhere in nature, and acidic polypeptides called silacidins [14]. Combinations of these organic molecules can associate via electrostatic interactions and precipitate silica *in vitro* in a variety of nanoscale morphologies, some of which resemble features of diatom silica[9]–[14]. The silica structures formed *in vitro* lack the degree of complexity of mesoscale silica structure found in diatoms [15], suggesting that other cellular components are involved. The intermediate scale of structure formation in diatoms is the mesoscale, which consists of structures of a few hundreds of nanometer size that are assemblies of the smaller building blocks of the nanoscale. The vast majority of distinct shapes that different diatom species make occur on the mesoscale, however, virtually nothing is known about the organic components that are involved in their formation. A recent study has suggested that chitin could be used as a template for the deposition of the silica [16], however, given the assembly properties of chitin, its growth would still require control by an additional component.

The largest scale of diatom silica structure formation is the microscale, which represents the final 3 dimensional shape of the frustule. This has been shown to be under the control of either active or passive shaping of the SDV by cellular organelles and the cytoskeleton [6]. Silica structures have been seen to mold around objects in the cell such as mitochondria [17], indicating that the SDV membrane (the silicalemma) can be shaped by external forces. In addition, a number of inhibitor studies and direct microscopic observations have shown that microtubules and actin play important roles in shaping microscale silica structure [18]–[20]. Addition of microtubule and actin assembly inhibitors has resulted in aberrations of meso- to micro-scale diatom silica structures [18]–[20], indicating an involvement by the cytoskeleton at these scales. TEM studies done by Pickett-Heaps and coworkers reported the presence of electron dense organic components with the appearance of microtubules or actin associated with the SDV during diatom valve morphogenesis [21]–[26]. In some cases, the organic components were associated with specific substructures, and it was suggested that the cytoskeleton was involved in shaping of the SDV during valve formation or inhibition of silica structure growth at particular locations. In spite of the high resolution possible by TEM, the nature of the organics were only inferred by their appearance, and TEM also does not enable evaluation of larger scale three dimensional relationships between silica and the organics. Another imaging option is fluorescence microscopy of the cytoskeleton, which lacks the high resolution possible by TEM, but enables positive identification of the components and reconstruction of patterns of assembly in three dimensions in a whole-cell context. Surprisingly, there have been only three previous reports visualizing the cytoskeleton in diatoms during frustule formation using fluorescence microscopy, and only the most recent one also stained for silica to allow correlation between the cytoskeleton and valve structure. Visualization of actin filaments and microtubules during valve formation in *Proboscia alata* and *Rhizosolenia setigera* [25]; [26], revealed an actin ring associated with the front of silica deposition in both species, and microtubules also associated with the expanding valves. More recently, actin was visualized in *Cyclotella cryptica*, identifying an actin ring that defined the full extent of the valves and another concentration of actin filaments associated with the growing front of silica deposition [27].

In other cell types, whole cell fluorescence microscopy has provided unprecedented insights into the dynamic roles that actin and microtubules play in shaping the cells and positioning components within them [28]; [29]. To clarify the role of the cytoskeleton in forming diatom silica structures, in this study we investigate actin and microtubule arrangements relative to forming silica, using fluorescence microscopy, SEM, and AFM. We compared 5 different diatom species from divergent classes, with distinct structures and shape to enable analysis of conserved and divergent roles of the cytoskeleton. The resulting three-dimensional whole-cell views indicate that the arrangement and dynamics of microtubules and actin are the major contributors to meso- and micro-scale patterning of silica in diatoms.

## Results

### *Coscinodiscus granii*

**Figure 1:**
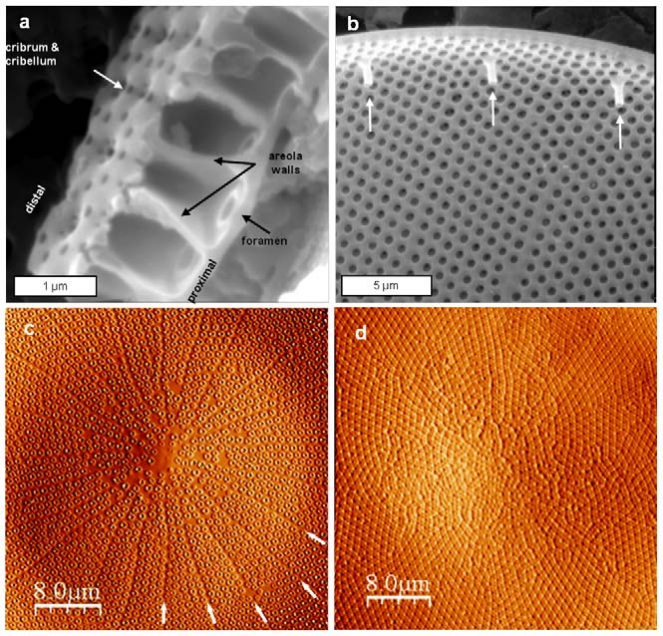
Structural arrangement of the valve of *Coscinodiscus granii*. a and b) SEM images; a) section view of the valve, b) proximal surface with rimoportulae at the edge. c and d) AFM images in deflection mode in the center of the valve; c) proximal surface, d) distal surface.

*Coscinodiscus granii* is a large centric diatom with a multilayer valve and tubular structures called rimoportulae localized around the rim. Valve formation in another *Coscinodiscus* species, *C. wailesii*, was described in detail previously [30], and our observations suggest a similar process of formation in *C. granii*. The valve is made of 3 layers formed sequentially, a flat base layer defining the proximal surface of the valve containing large pores called the foramen, followed by formation of chambers in the structure called areolae that are built of side walls surrounding the foramen, and then followed by formation of a layer of pores called the cribrum and even smaller pores called the cribellum that constitute the distal surface of the valve (Fig. 1). A cutaway view of the arrangement of these structures is shown in Fig. 1a. Atomic force microscopic images show the overall structure of the proximal and distal valve surfaces (Fig. 1c and d). An interesting observation on the proximal valve surface was the superposition of a linear, radial structural pattern over the general pattern of the foramen (Fig. 1c, arrows).

**Figure 2:**
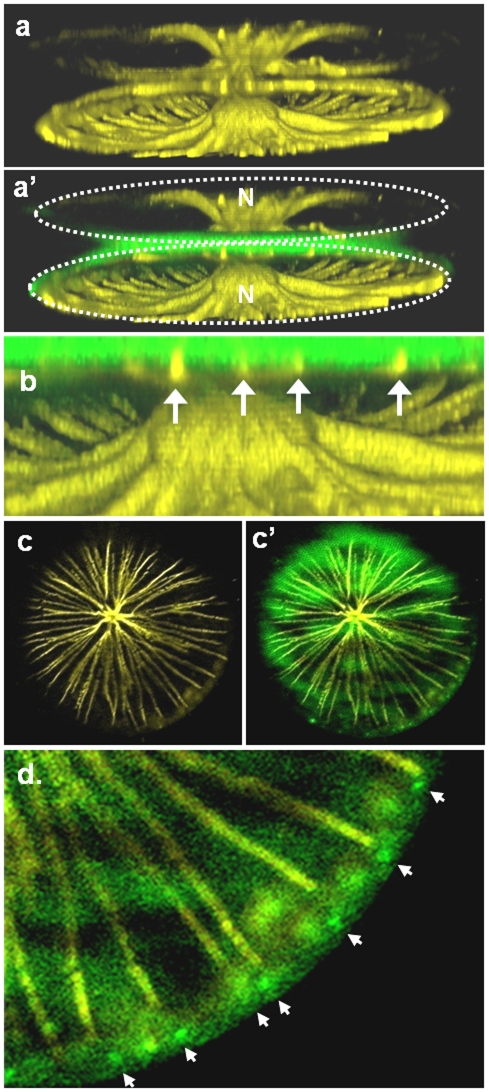
Localization of microtubules in forming valves of *C. granii* (microtubules in yellow, silica in green). a, a′) Girdle-band plane view of a 3D reconstruction of a dividing cell, dots outline the overall position of the 2 daughter cells. b) zoom in the area of valve formation showing microtubules in contact with the forming valves. c, c′ and d) top view (z stack) in the center of a dividing cell at the site of valve formation. In (d) the proximity of microtubules to rimoportulae (arrows) is highlighted. Scale bar 20 µm.

We examined the association of microtubules with the developing valve by fluorescence microscopy, using PDMPO to stain the newly formed silica, and a tubulin-specific antibody to stain microtubules (Fig. 2). A girdle band-plane view of a cell in the process of new valve formation (Fig. 2a and a′) shows several interesting features. There is an extensive association between microtubules and the previously formed mother cell valves that is centered around the nucleus (Fig. 2a′). Examination of this and other images indicated that a denser microtubule network was associated with the epitheca than the hypotheca. We observed a close association between microtubules and the proximal surface of the newly forming valve which is stained by PDMPO (Fig. 2b - arrows). A branched and radiating microtubule structure was visible from the valve-plane view of the central portion of a cell undergoing new valve synthesis (Fig. 2c and c′). The microtubule arrangement closely resembled the pattern of the superimposed line structures on the proximal surface of the completed valve seen in Fig. 1c. The ends of the microtubules were located in close proximity to the rimoportulae (Fig. 2d, arrows).

**Figure 3:**
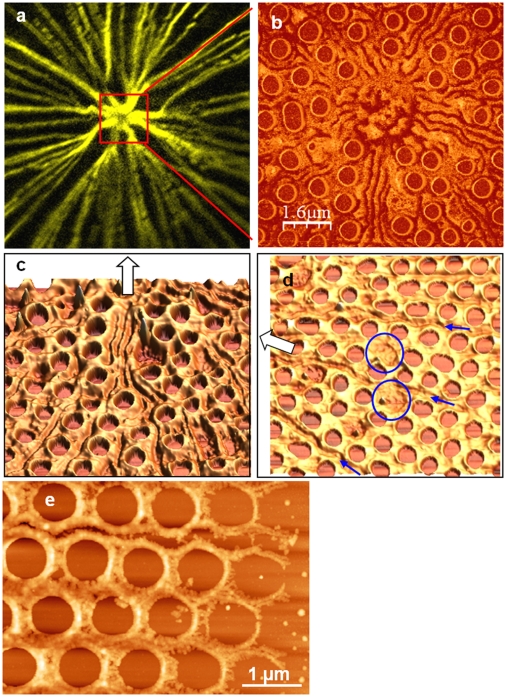
Visualization of indentations made in the proximal valve surface of *C. granii* by microtubules. a) Microtubule network associated with the forming valve, red square represents a similar area imaged by AFM in (b) in a different cell. b) AFM picture in phase mode in the center of an immature valve. c–e) AFM pictures in height mode of immature valves (c and d = 8 µm width) from the area next to the center of the valve (c) to the edge of the valve (e). White arrow indicates the direction to the center of the valve, blue arrows and circles indicate indentations left by microtubules and chloroplasts, respectively.

Examination of the proximal valve surface by AFM revealed linear indentation patterns superimposed over the base layer that correlated with the microtubule pattern (Fig. 3). At the center of the valve, these lines converged to resemble the core of the microtubule center (Fig. 3a and b). Multiple adjacent lines were observed nearer the valve center (Fig. 3b and c), which branched and eventually became single lines towards the valve rim (Fig. 3d and e), as was observed for the microtubules (Fig. 3a). The width of single indentations was around 300 nm and 20 to 40 nm deep. On the fluorescent micrographs the apparent width of the microtubule staining at the periphery at the limit of resolution was around 500 nm. Since individual microtubules are known to be 24 nm in diameter, the indented features on the *C. granii* valve likely represent microtubule bundles. The lines were present prior to silica deposition, as can be seen by observation of the silicification front in Fig. 3e, where a gap between adjacent sets of foramen is visible. Because of the correspondence in detailed pattern and size between the lines and microtubules, we hypothesized that they are formed by microtubules indenting the surface of the SDV which in turn indented the forming silica. We also observed roughly circular imprints represented by featureless areas where foramens were absent (Fig. 3d, circles). Based on fluorescence imaging of the pattern and size of chloroplasts (data not shown), we believe that these imprints were due to chloroplasts pushing on the SDV during valve formation.

**Figure 4:**
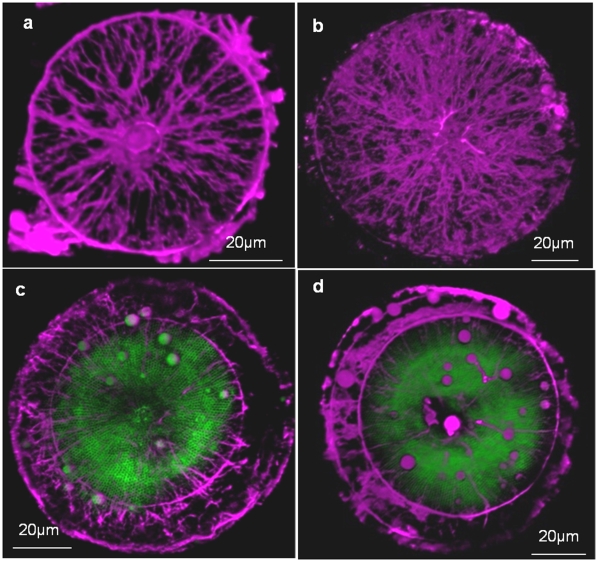
Actin (purple) and silica (green) localization from the valve view in forming valves of *C. granii*. a) Actin network associated with the forming valve at an early stage, the actin ring defines the edge of growth and has not yet expanded to the periphery of the cell. b) Actin network associated with the forming valve at later stage. Comparing a and b shows an increase in the actin network. c and d) Actin and silica in the area of valve formation at early stage. In both images, the ring in one daughter cell is smaller than the other, demonstrating that expansion of the ring occurs (compare c with d).

We next examined the location of actin filaments in relation to forming valves. Similar to what was observed in *Cyclotella cryptica* [27], a prominent actin ring defining the edge of the SDV was present, along with a filamentous actin network that filled the ringed area (Fig. 4). The filamentous network consisted mostly of a radial alignment of actin, and as valve formation progressed the amount of actin increased (compare Fig. 4a and b). The actin rings expanded as the valve formed (Fig. 4c and d), as was seen previously in *C. cryptica* [27].

**Figure 5:**
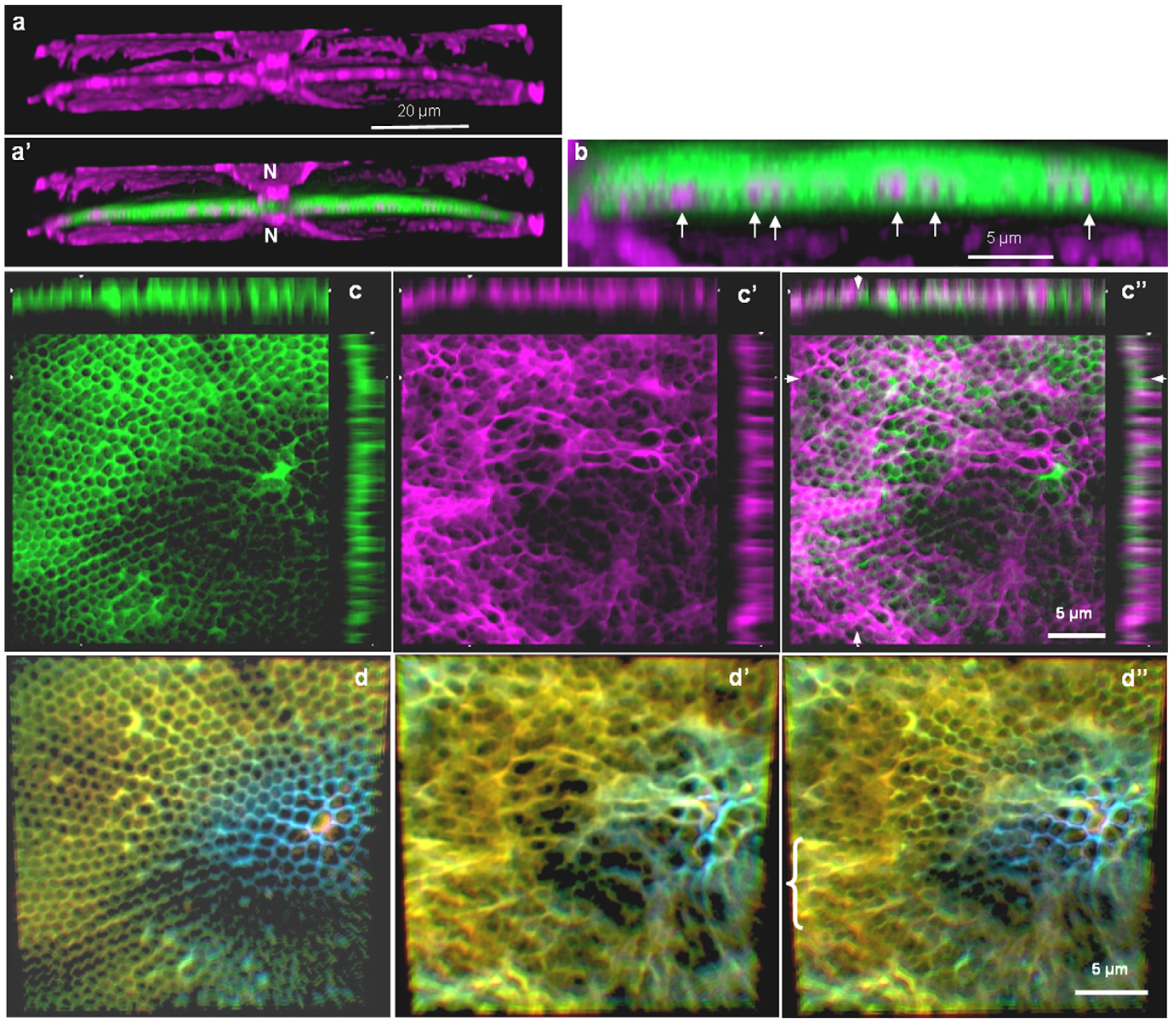
Correlation between actin (purple) and mesoscale silica structure (green) in forming valves of *C. granii*. a, a′) Girdle band-plane view of a 3D reconstruction in the center of a dividing cell showing the association of actin with the mother cell valves, the nucleus (N), and the forming valve. b) Interdigitation of actin (arrows) and forming silica. c–c″) A single optical slice showing silica (c), actin (c′) and silica plus actin (c″). Optical slices of the images are projected at the top and right of each, the location of the slice is denoted by arrows. d–d″) 3 dimensional reconstruction with depth color coding showing silica, actin and a composite of both, respectively in a forming valve. Bluer tones are further away in the z-axis direction, yellower tones are closer. Bracket in d″ locates a region where the pattern of actin does not correlate with the silica.

Fig. 5a and a′ is a girdle band-plane view that shows an association between actin and the mother and daughter cell valves, which also surrounded the nucleus. In newly forming valves, some actin filaments appeared to be interdigitated within the silica (Fig. 5b, arrows). Using an optical sectioning approach on a valve-plane-view image, we confirmed the interdigitation in Fig. 5c″. Actin completely spanned the silica in some locations, and extended into the cytoplasm on the proximal surface of the valve (Fig. 5c″).

In Fig. 5c and d we observe the association between actin and newly formed silica in the valve-plane view. In addition to the interdigitation, we see correspondence between the pattern of actin and the outlines of the foramen (Fig. 5c–c″). There are regions where the actin filaments do not strictly follow the pattern of the foramen – there are regions where less conforming actin filaments are observed, and appear to be in a different plane than the valve surface. The combination of both closely associated and less associated actin can be seen more clearly in a 3 dimensional reconstruction with depth color coding (Fig. 5d–d″). These data (Figs. 5) indicate that a portion of the actin network is intimately associated with, and shares a similar pattern to, the foramen structure, and that this portion is connected to an actin network that extends into the cytoplasm.

**Figure 6:**
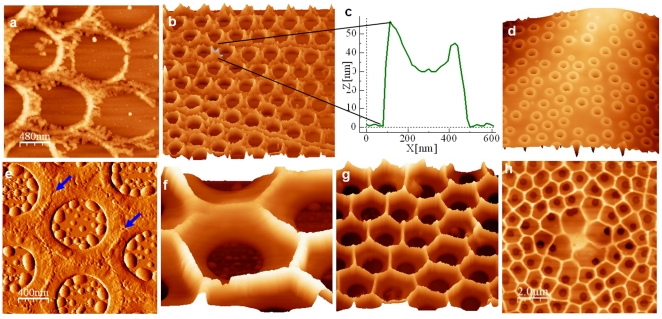
AFM images of different stages during *C. granii* valve formation. a, b, d, f–h are height mode, c is a height profile and e is in deflection mode. a) Initial deposition of silica as particulate thin filaments in the outline of the foramen. b) The forming front of the base layer, showing initially deposited silica towards the right, with increasing maturation towards the left. Scan size is 8 µm. c) plot of height profile in the denoted section of (b). d) Completed base layer from the proximal side showing the foramen. Scan size is 10 µm. e) Initial formation of the walls of the areolae. The spaces between the initial silica strands that define the foramen are filled in, making a circular structure, and the walls of the areolae begin to form where the circles overlap (arrows). f) Formation of the areolae walls at an initial stage. Scan size is 1.7 µm. g) A later stage showing peaks that occur at the junction of three adjacent walls. Scan size is 5 µm. h) Pattern of the areolar walls from the distal view prior to cribrum formation. The partially irregular arrangement is apparent, showing that the walls are defined by the positions of adjacent foramen. Scan size is 10 µm.

As in *C. wailesii* [30], formation of the base layer and foramen in *C. granii* involved radial growth of thin silica ribs from the valve center, and as the ribs extended, branching occurred on adjacent ribs from which thin silica structures grew towards each other to form cross-connections, which are the basic outline of the foramen, as seen in the proximal-surface view in Fig. 6a. The foramen structure becomes raised relative to the base layer, and intermediates show an unequal growth around the sides (Fig. 6b and c). Eventually, the irregular structure becomes filled in, forming a relatively smooth surface between the raised rims of the foramen (Fig. 6d). After completion of this layer, the walls of the areolae begin to form on the distal side. At the initial stage, rings of silica were deposited around the central opening, and raised ridges were formed where adjacent rings were appressed (Fig. 6e). These ridges increased in height while growing perpendicular to the base layer, and generated common boundaries between adjacent areolae (Fig. 6f–g). The extent of growth was not uniform across the valve, but was more advanced near the center and less advanced radially towards the edges. The relatively regular pattern of the foramen is not maintained in the areolae – the walls of an individual areola are not necessarily equal length, and since they form by fusion of adjacent walls, the structure becomes irregular, especially in the center because there are no underlying foramens in this region (Fig. 6h).

**Figure 7:**
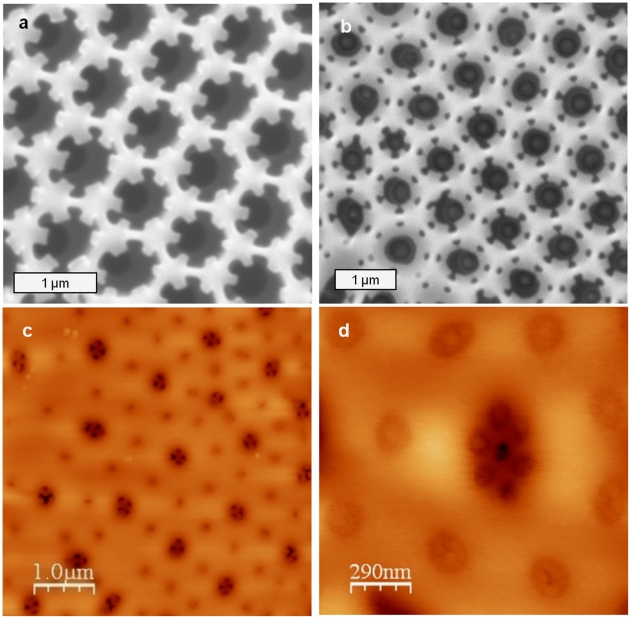
Cribrum and cribellum formation in *C. granii*. a) SEM of the initial stage of cribrum formation, triangular silica structures formed at the junction of three areolar walls are visible. b) SEM of a later stage of cribellum formation. The outer hexagonal arrangement of cribella adjacent to the areola walls has formed, but the inner pore has not. c and d) AFM (height mode) showing the arrangement of pores in the cribellum.

Formation of the cribrum was initiated at the junction of three adjacent areolar walls, and manifested by the formation of a triangular silica structure, with the apices of the triangle projecting in towards the open center (Fig. 7a). These structures grew towards the center and generated a distinctive generally hexagonal pore arrangement near the rim of the areolae, and a larger central pore with a central opening flanked by a hexagonal arrangement of the cribellum (Fig. 7c–d).

### Examination of Other Species

The extensive correspondence between the cytoskeletal components and silica in *C. granii* suggested that actin and microtubules are major spatial control elements of silica formation on the meso- and micro-scale in diatoms. *Coscinodiscus* species are a distinct class of diatoms that tend to have a larger size than most other classes, and have the specific three-dimensional structure described above. A valid question is whether the roles of actin and microtubules are similar in smaller species, or those with diverse structures. For this reason, we examined other species with distinct silica structures to determine the relationship between the cytoskeleton and forming silica.

### *Surirella* sp

**Figure 8:**
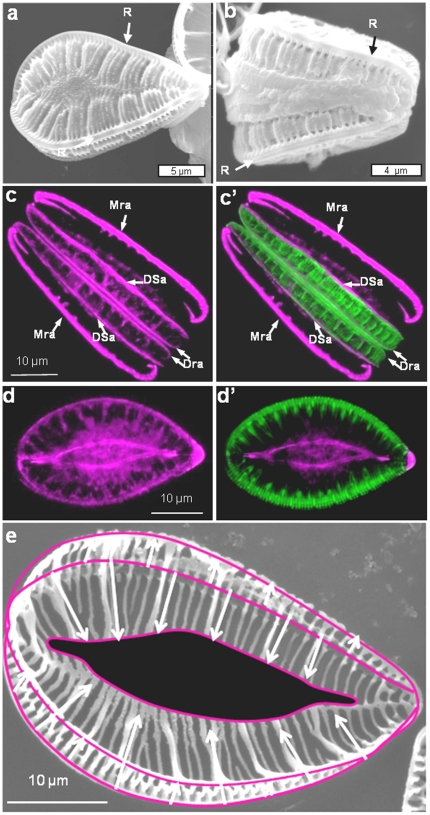
Actin localization during valve formation in *Surirella* sp. a) SEM of a valve plane view of *Surirella* sp. R locates the raphe. b) SEM of a girdle band-plan view of Surirella sp. c and c′) Actin (left) and actin and silica (right) localization in a girdle band-plane view of *Surirella* sp. Mra  =  mother cell raphe actin, Dra  =  daughter cell raphe actin, DSa  =  daughter cell SDV actin. d and d′) Valve plane view localization of actin and silica in *Surirella* sp. e) Schematic superimposed on an SEM of a forming valve showing the bidirectionality of growth from the raphe.

**Figure 9:**
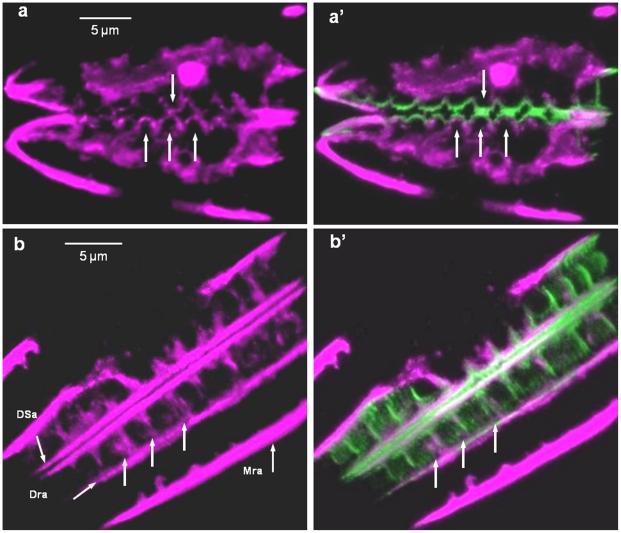
Correlation between actin and silica patterning of mesoscale features on valves of *Surirella* sp. a and a′) Correlation between scalloped features on the valve edge, actin (a) and actin plus silica (a′). Arrows locate prominent scallops. b and b′) Correlation between rib structures. Mra  =  mother cell raphe actin, Dra  =  daughter cell raphe actin, DSa  =  daughter cell SDV actin. Arrows locate prominent ribs in which actin and silica correlate.

A unique feature of the valve architecture of *Surirella* species is that they contain a raphe along the entire edge of the raised perimeter of the valve (Fig. 8a and b). Valve formation starts at the raphe along the periphery of the cell. Actin localization in a girdle-band view of a cell undergoing new valve synthesis revealed a prominent actin band associated with the mother cell raphe (Mra – Fig. 8c and c′), another actin band at the location of the daughter cell raphe (Dra), and yet another actin band defining the periphery of the daughter cell SDV (DSa) as ribs form from the daughter cell raphe. After formation of the raphe, silica ribs grew simultaneously from the raphe towards the center of the valve and from the raphe to the side of the cell to form the lower rim (Fig. 8c–e). A valve-plane view shows another concentration of actin in the center of the valve which defines another edge of the SDV (Fig. 8d and d′). The periphery of the valve is undulated, and there is correspondence between undulations in the newly deposited silica and in actin (Fig. 9a and a′). We also see a correspondence between actin and forming ribs on the side of the valve (Fig. 9b and b′).

### *Nitzshia curvilineata*

**Figure 10:**
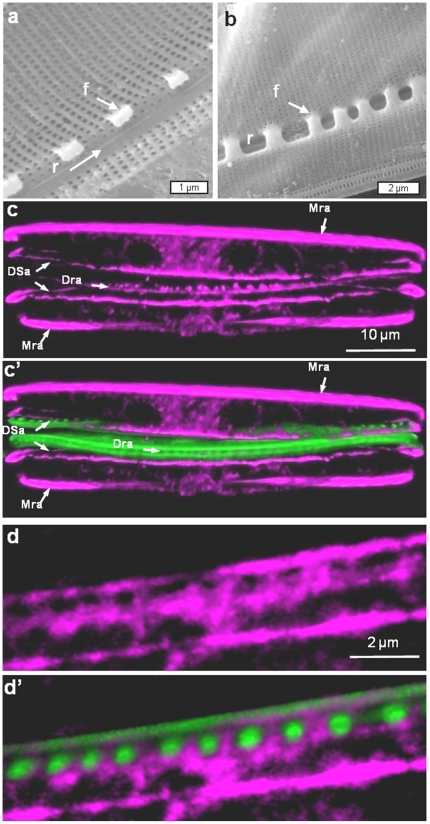
Raphe formation in *Nitzschia curvilineata.* a and b) SEM of forming raphe showing and earlier (a) and later (b) stage of development of the fibulae which span the raphe fissure; r =  raphe, and f =  one of several fibulae. c and c′) Actin and silica localization during raphe formation. c) Actin alone, c′) Actin plus silica. Mra  =  mother cell raphe actin, Dra  =  daughter cell raphe actin, DSa  =  daughter cell SDV actin. d and d′) Higher magnification image of actin and silica localization during raphe formation. d) Actin alone, d′) Actin plus silica. Actin is seen to completely surround the silica.

Valve formation in *N. curvilineata* starts with the formation of the raphe on one side of the valve, followed by growth of silica ribs on each side. In this species, the raphe is a two-part structure which is connected by struts called fibulae that prevent the two halves from splitting. The growth and structure of fibulae is shown in Fig. 10a and b. Localization of actin in a cell forming a new raphe shows actin associated with the mother cell raphe (Fig. 10c and c′ – Mra), actin associated with the daughter cell raphe (Dra), and actin associated with the expanding SDV (DSa). Close examination of newly-formed fibulae shows that actin completely surrounds the silica of these structures (Fig.10d and d′).

### *Entomoneis alata*

**Figure 11:**
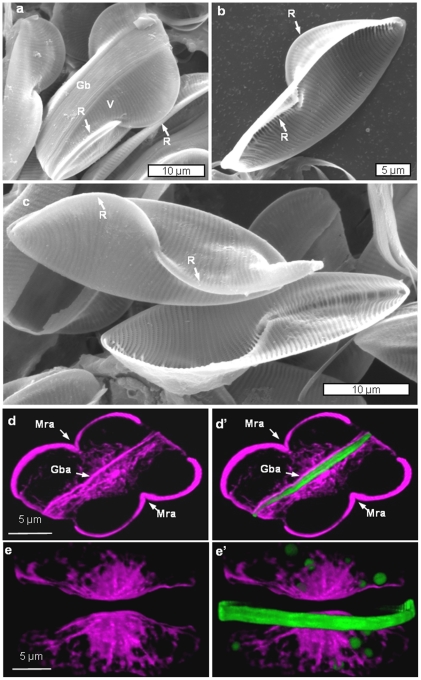
SEMs of frustule structure of *E. alata* and actin and silica during and after girdle band formation in *E. alata*. a) Intact frustule of *E. alata*. V =  valve, gb  =  girdle band, R  =  raphe. b) A single valve of *E. alata*. c) Two valves of *E. alata*. d and d′) Actin and actin plus silica, respectively, during girdle band formation. Mra  =  mother cell raphe actin, Gba  =  girdle band actin. e and e′) Actin and actin plus silica after girdle band formation but prior to valve formation showing dynamic rearrangement of the actin network.

The valve structure of *E. alata* is quite unique, and is dominated by two keels that contain the raphe along their mid-lines (Fig. 11). After addition of silicon to a silicon-limited culture of *E. alata*, the first structure formed is a girdle band (Fig. 11). Actin localization in Fig. 11 reveals the mother cell raphe actin (Mra) and a relatively heavy actin filament associated with one side of the forming girdle band (Fig. 11d and d′). During mitosis, we see a rearrangement of the actin no longer associated with the labeled girdle band (Fig. 11e and e′).

**Figure 12:**
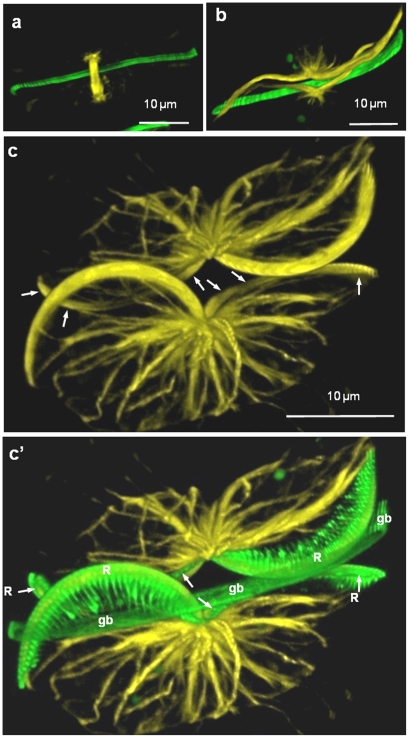
Microtubules and silica during mitosis and raphe formation in *E. alata*. Microtubules are yellow, silica is green. a) Microtubule spindle during mitosis. The previously labeled girdle band is visible. b) Reorganization and expansion of microtubule network after mitosis. Microtubules are forming the shape of the keel and are also networked into other regions of the cell. c and c′) Microtubules and silica during raphe formation. Arrows locate the position of the less visible portion of the keel. R =  raphe, gb  =  girdle band.

We examined the arrangement of microtubules in Fig. 12. Fig. 12a shows a cell in metaphase or anaphase with the mitotic apparatus visible along with the labeled girdle band. Fig. 12b shows the microtubule arrangement after cytokinesis, but prior to silica deposition. At this stage microtubules are visible forming the keel-shaped arrangement that they will later assume during raphe formation. Raphe formation is documented in Figs. 12c and c′. Visible are the predominant keels as well as the less visible ones offset at an angle (arrows). The backbone of the raphe has formed and ribs are emanating from them (Fig. 12c′). Microtubules also form an extensive network throughout the remainder of the cell (Fig. 12c).

**Figure 13:**
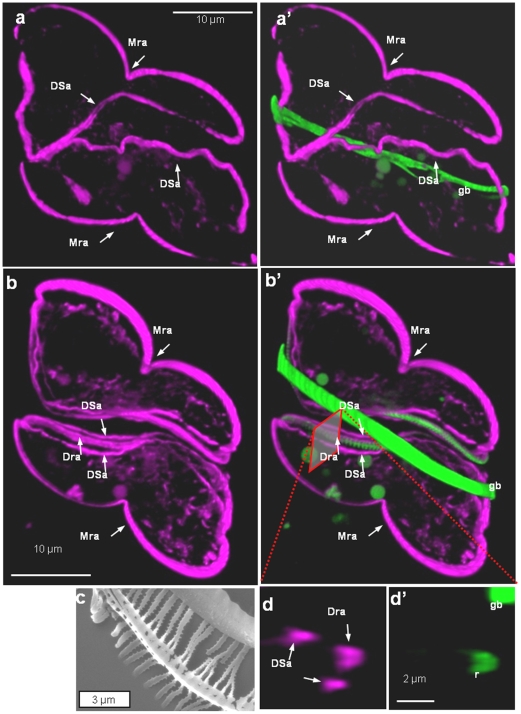
Actin and silica organization during raphe formation in *E. alata*. Mra  =  mother cell raphe actin, Dra  =  daughter cell raphe actin, DSa  =  daughter cell SDV actin. a and a′) Actin (left) and actin plus silica (right) of a cell which has not begun raphe synthesis, but has organized actin in preparation for it. b and b′) Actin (left) and actin plus silica (right) of a cell undergoing raphe synthesis. c) SEM of ribs forming from the raphe. d and d′) Actin (left) and actin plus silica (right) of the windowed area of (b′) distinguishing between actin associated with the forming raphe (Dra) and the SDV (DSa).

Actin arrangement is visualized in Fig. 13. Prior to raphe formation (Fig. 13a and a′), we see the mother cell raphe actin (Mra), the labeled girdle band, and what appears to be SDV actin that will be associated with the daughter cell raphe (DSa). During raphe formation (Fig. 13b and b′), in addition to the daughter cell SDV actin, a thicker actin filament directly associated with the raphe (Dra) is visible. After raphe formation, ribs are formed radiating from the raphe (Fig. 13c) actin filaments have been observed associated with these ribs (data not shown) and the SDV defines the edge of rib growth. The actin/SDV arrangement is seen in a cross-sectional image, where the raphe and SDV actin are visible, but only silica deposition at the raphe. The shape of the raphe and actin correspond to each other (Fig. 13d and d′).

### *Triceratium dubium*

**Figure 14:**
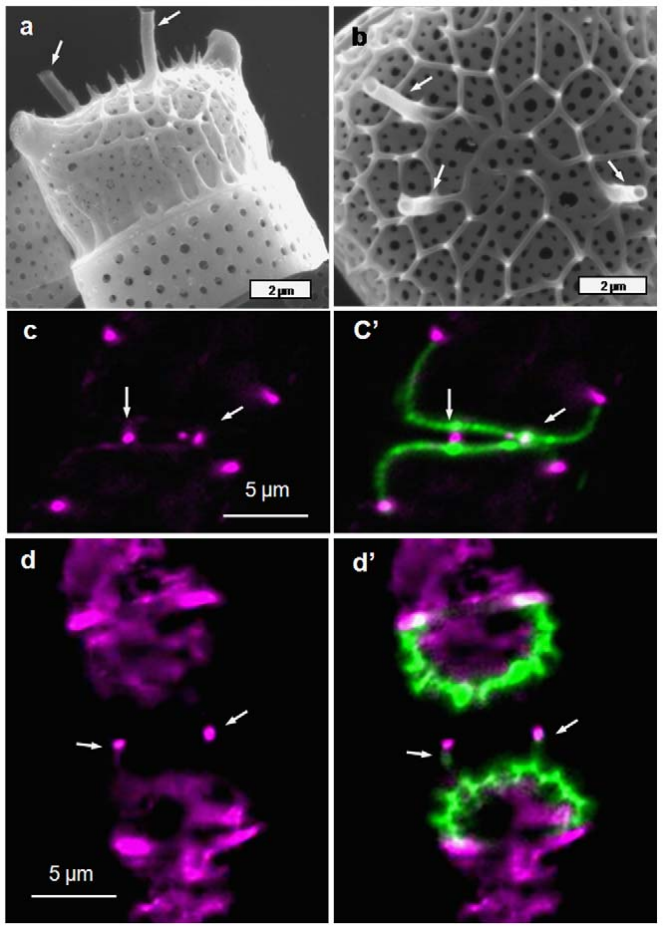
Actin association with the tips of rimoportulae in *Triceratium dubium*. a and b) Rimoportulae (arrows) on the valve. c and c′) Actin and silica in two cells of T. dubium. Arrows locate actin at the tips of the rimoportulae. d and d′) Actin and silica in cells of *T. dubium* in which the tips of the rimoportulae have grown relative to c and c′.

*Triceratium dubium* possesses tube-like structures called rimoportulae that are found in varying numbers towards the center of the valve (Fig. 14a and b). Previous examination of tubular structures called setae in *Chaetoceros* by TEM suggested the presence of an actin collar at the tip of the growing setae, but this could not be confirmed by fluorescence labeling due to low intensity [24]. In *T. dubium*, in addition to visualizing actin associated with valve formation, an intense fluorescence at the tips of the rimoportulae was visible, precisely where predicted in the setae of *Chaetoceros* (Fig. 14c and c′). The actin remained associated with the tips as they continued to grow (Fig. 14d and d′).

## Discussion

By both confirming previous observations and extending them with new information, this study demonstrates that actin microfilaments and microtubules play a predominant role in meso and micro-scale frustule morphogenesis in diatoms. The unique features of the current study are 1) a correlation between silica structure and the cytoskeletal elements by dual fluorescence staining, 2) three dimensional reconstruction of large-scale features, in some cases using optical slices, and 3) examination of cytoskeletal elements in a diverse variety of diatom silica structures.

Many inhibitor studies have been performed on diatoms showing that both microtubules and actin play important roles in structure formation [18]–[20]. In terms of microscopic evaluation, TEM studies done by Pickett-Heaps and coworkers reported the presence of electron dense organic components with sometimes the appearance of microtubules or actin filaments associated with the SDV during diatom valve morphogenesis [21]–[26]. Based on their relative arrangements, the organic components were suggested to shape the SDV during valve formation or inhibit the growth of silica at particular locations. Although TEM allows high resolution evaluation of the interaction between the organic components and silica, the nature of the organics are only inferred by their appearance (which is especially challenging with actin - often described as “fibrous material”), and TEM lacks the ability to correlate larger scale three dimensional relationships between silica and the organics, limiting interpretation of their roles. Only three previous reports analyzed the role of the cytoskeleton during frustule formation using fluorescence microscopy [25]–[27], and only the most recent one also stained for silica to allow observation of the cytoskeleton and valve structure [27]. The results presented here clarify the role of the cytoskeleton in diatom silicification in a whole cell context.

### Microtubules

We examined microtubules in two contexts, 1) valve formation in the centric diatom *Coscinodiscus granii* (Fig. 2), and 2) raphe formation in the pennate diatom *Entomoneis alata* (Fig. 12). The antibody-based microtubule staining approach did not work on all species, hence a more extensive survey was not possible. Microtubule involvement in centric valve formation has not been well documented [6]. In *C. granii*, microtubules were visualized as thick bundles around the center of the valve which branched as they radiated to the edges of the valve (Fig. 2c and c′). Given the limit of resolution of fluorescence microscopy, only bundles could be visualized – it is therefore possible that the microtubule network is more extensive than seen here. The microtubule organization is correlated with the indentation pattern observed by AFM on the proximal valve surface (Fig. 3). Similar indentations were observed in previous studies in *Coscinodiscus* sp. [30]–[32]. A cross-sectional view shows that microtubules are in contact with the valve but do not extensively interdigitate with the silica (Fig. 2b), however the extent of indentation observed (Fig. 3) indicates that the microtubules and SDV exert pressure against each other. We also observed some correlation between portulae location and the tip of the microtubules (Fig. 2d). This observation is in accordance with results in *C. cryptica* showing that a microtubule inhibitor affects positioning of portulae [27]. In a study of *D. brightwellii*, it was shown that the cell uses microtubules attached to the portulae as spatial cues during recovery after plasmolysis [33].

Observation of the microtubule arrangement in a whole-cell context in *C. granii* shows that it is a highly organized network centered on the nucleus (Fig. 2a). Previous TEM examination of *C. wailesii* suggested that the nucleus was in contact with the forming valve [30]; our results suggest that in *C. granii*, the nucleus remains more closely associated with the epivalve (Fig. 2). Microtubule filaments run from the periphery of the epivalve, around the nucleus until the edge of the newly forming hypovalve (Fig. 2a and a′). Spatial positioning of not only the nucleus, but of the SDV during division is probably critical especially for a large cell like *C. granii*. Other studies in pennate diatoms have documented the role of microtubules in positioning the nucleus, and in moving initially formed silica structures during the process of new valve formation [17]; [34]; [35]. It is possible that the microtubule network is used by the cell to position silica structures of the new valve relative to the positioning of those of the old valve. This mechanism could explain the phenomenon of “interactive division” where the two forming valves have complementary shapes [36]; [27]. More precise mapping of the microtubule arrangement in epi- and hypo-valves is required to substantiate this, but it seems a plausible mechanism.

In pennate diatoms, microtubules have previously been shown by TEM to be associated with the forming raphe; however their precise role in shaping that structure is still unclear [6]. The use of microtubule-disrupting drugs results in the formation of an abnormal raphe and a partially occluded raphe fissure suggesting an essential role [6]. In *E. alata* we observed a substantial assembly and reorganization of microtubules after cytokinesis at the site of future raphe formation (Fig. 12). The raphe-associated microtubule bundle was formed before any silica deposition occurred (Fig. 12b) and appeared to pre-form the shape for the future raphe keels (Fig. 12c and c′). The prepositioning of the microtubules suggests an important role defining the site of SDV positioning and formation.

In previous studies, microtubules were seen associated with silica ribs in different species [24], but our results suggest that they are not necessarily involved in positioning of ribs or in their growth. In *C. granii*, the indentation patterns on the proximal valve surface show that microtubules are distinct from the initially deposited ribs of the base layer (Fig. 3). In *E. alata*, although microtubules define the position and shape of the raphe, they are not visibly associated with the perpendicular ribs growing from the raphe (Fig. 12c and c′).

### Actin Filaments

Our results indicate that actin filaments play a substantial role in formation of meso- and micro-scale structure in diatom frustule formation. One role is in defining microscale processes such as the size and shape of the SDV and the edge of the front of silicification. In *C. granii* (Fig. 4) and in previous work on other centric diatoms [25]–[27] a prominent actin ring was identified that defined the front of silicification, and by inference, the edge of the SDV, which increased in diameter as the valve expanded. In the pennate species, we consistently see actin defining the edge of the SDV, but also another actin band associated with the forming raphe (Figs. 8, 10, and 13). Due to the unique structure of valves in *Surirella*, we see two actin “rings” associated with expansion of the SDV over the surface, and along the sides of the valves (Fig. 8). Actin is also involved in girdle band formation - in *E. alata* we see a band of actin associated with a forming girdle band, and in a previous study actin-sized filaments were observed associated with a forming girdle band in *T. pseudonana* [37].

In addition to actin's role in microscale processes, it is also involved in shaping the formation of mesoscale structures such as ribs and other features. In *C. granii*, we observed a correlation between the actin network inside the outer ring and the mesoscale silica structure of the foramen in the base layer (Fig. 5). The density of the actin network increased as the valves expanded (Fig. 4). The correlation between actin and foramen patterning suggests that actin defines the foramen by positioning the deposition of the thin ribs in the base layer (Fig. 6). In *C. granii*, we also see that actin interdigitates within the silica (Fig. 5). The only area in which this could occur is in the chambers of the areolae, suggesting that actin could be involved in z-axis growth of the walls of the areolae chambers and positioning of the distal layer of the cribrum and cribellum. In *T. eccentrica*, cross-sections of the areolar chambers show that the silicalemma completely surrounds the growing areolar walls, leaving the central portions of the chamber essentially exposed to the cytoplasm through the opening of the foramen [38]. Thus, a reasonable explanation for actin's interdigitation with the *C. granii* valve is that it passes through the foramen and could either directly interact with the silicalemma that is associated with the areolar walls, or simply enable spatial relief between the SDV and plasmalemma to accommodate the z-axis expansion.

A close correspondence between the position and shape of actin filaments and ribs or scalloped structures in *Surirella* (Fig. 9), fibulae in *N. curvilineata* (Fig. 10), and the raphe of *E. alata* (Fig. 13) are all consistent with these filaments being responsible for patterning the silica deposition process at the mesoscale.

### Actin, microtubules, and the raphe

In the pennate diatoms, actin is associated with the mother cell raphe as part of the cellular motility mechanism [3], however, no evidence of its presence during valve formation has been reported. We show that actin is a critical component of raphe and rib production (Fig. 8, 10, 13). It is unclear what the relationship is between actin involved in raphe formation and actin involved in motility, and whether the latter is derived from the former. In raphe formation, the first observable actin structures are filaments defining the SDV, and actin associated with the raphe appears later when silicification begins (Fig. 13). In section, the actin filament associated with the raphe of *E. alata* has the same v-shape as the silica (Fig. 13d and d′), in accordance with a role of shaping of the silica.

### Rimoportulae and tip growth of tubular structures

In *T. dubium*, actin was observed at the tips of the rimoportulae, and remained at the tip regardless of their length (Fig. 14). This suggests that actin is continually associated with the tip as the portulae grow. During formation of tubular silicified spines called setae in *Chaetoceros*, Pickett-Heaps described a fibrous band lining the internal surface of the silica at the growing end of the spine [39] This structure was proposed to be actin, and a model for its involvement in tip growth was presented [39]. It was proposed that the actin band defined the diameter of the seta, and that it was propagated along the growing tip either as an intact unit via a molecular motility system, or by a treadmilling mechanism in which actin monomers from the base of the band were recycled at the tip [39]. In other diatom species, another tubular structure called the labiate process exists. In a study of *R. setigera* morphogenesis, van de Meen and Pickett heaps [26] also noticed a fibrous plug at the tip of the labiate process. Cytochalasin D, a drug that disrupts actin filaments, inhibited elongation of the tube, suggesting that the fibrous plug at the tip could also be actin. The fluorescence images in Fig. 14 positively confirm the presence of actin at portula tips in *T. dubium*, and when combined with previous observations, these data suggest a common mechanism of tip growth in some types of diatom tubular structures, consistent with models previously postulated [39].

### The role of the cytoskeleton in forming SDV structure

It is useful to consider the physical properties of the cytoskeleton, and of microtubules and filamentous actin specifically, in evaluation of their roles in shaping diatom silica structure. Cytoskeletal components enable long-range order in a cell, and considering the size of silica structures that are formed in diatoms, and the control over long distances required for their formation, it is logical that they would be important components of diatom silicification. Microtubules are stiff polymers that assemble to form generally linear structures in a directional manner [29]. They are useful for positioning components within the cell, with a classical example being chromosome segregation during mitosis, in which this positioning has a dynamic aspect. Actin is generally involved in dealing with tension or compression forces in a cell and can form a wide variety of assembly patterns via the intermediary of other proteins that affect its assembly properties [29]. Both microtubules and actin can associate with membranes and affect their shapes, and both also are able to transport cargo along their lengths in a directional manner.

In the context of diatom silica structure formation, microtubules appear to be involved in the microscale positioning of components, and perhaps in maintaining tension within the SDV. Inhibitor experiments in other diatom species are consistent with this interpretation [18]–[20], as are previous fluorescence microscopy studies which suggested that microtubules are involved in the strengthening and overall shaping of the SDV on the microscale [25]; [26]. In *E. alata*, extensive microtubule arrangements define the shape of the keel even prior to silica deposition (Fig. 12), which is consistent with a role of positioning the SDV. In *C. granii*, microtubule bundles not only define the extent of the valve SDV, but apparently position the portulae (Fig. 2), which was also inferred in *C. cryptica* by the use of microtubule inhibitors [27]. The radial arrangement of microtubules in *C. granii* suggests that they may be responsible for extending the SDV to its full diameter. During silica polymerization, the SDV should be under substantial osmotic stress due to the formation of a solid phase within it, and perhaps the stiff microtubule structure can mitigate structural deformations that may occur. Due to the lack of correspondence between the microtubule patterns and mesoscale features in the diatom species observed here, it is not likely that microtubules play a substantial role in patterning of the silica structure at the mesoscale.

Our results indicate that not only do actin filaments contribute to microscale patterning, but they are the primary determinants of mesoscale silica structural patterning in diatoms. The general role of actin derived from this study is that of a membrane-definer. The actin rings observed in *C. granii* and previously in other centric diatom species [25]–[27] clearly define the edge of the SDV and dynamically expand as the structure grows. Actin is well known to participate in membrane stabilization [40]; [41] and such a characteristic is likely to be important in the deposition of the solid silica material adjacent to the SDV membrane. In addition to the ring structure defining the microscale, filamentous actin defines many of the mesoscale structures (Figs.9, 10, 13). Actin can also assemble in other patterns via interaction with other proteins that affect actin assembly properties, and the branching, circular, or scalloped structures seen here (Figs. 9, 10, 13) may be manifestations of this. The data are entirely consistent with actin being responsible for positioning of polymerization determinants within the SDV on the mesoscale.

In addition to assembling to form different structures, another possible role for actin is in membrane shaping. Actin is known to be involved in many membrane remodeling events like cytokinesis, exo and endocytosis, motility and cellular protrusion [40]; [42]. The cytoskeleton influences membrane shape by controlling membrane tension and interacting with specific proteins associated with the membrane [40]; [43]. In this case, rather than specifically positioning polymerization determinants within the SDV, actin could be shaping the silicalemma to provide a defined shape within which silica is precipitated. There is some data suggesting that confinement, rather than direct templating, does occur in diatom silicification [37].

The ability of both microtubules and actin to participate in cellular trafficking suggests another important role in diatom silica structure formation. The assembly of the SDV is a major cellular event in which all components of this complex organelle must be correctly positioned over a limited period of time. The presence of microtubule and actin networks that interface between the SDV and remainder of the cell (Fig. 2, 5, 12) provides a reasonable explanation for how this is accomplished – appropriate components could be trafficked along the cytoskeletal elements to specific locations in the growing SDV to form specific silica structures. In addition, the basic expansion of the SDV during valve formation (Fig. 4) could result from motor proteins trafficking along the microtubules.

### The role of the cytoskeleton and SDV in relation with the other components of silicification

It is clear that the cytoskeleton plays a substantial role in the formation of silica structures in various parts of the diatom cell wall. There are two important spatial aspects to consider in this. First is that microtubules and actin must function external to the SDV lumen – they are not known to directly cross membranes. The second is that microtubules and actin are likely to be limited to interaction with structures that are accessible from the cytoplasm (Fig. 2 and 5). Regarding the second point, this could include structures on a distal surface as long as that surface is accessible from the cytoplasm, as is apparent through the foramen in *C. granii* (Fig. 5) and *T. eccentrica* [38], but in most other centric diatom species, the base layer spans the entire diameter of the cell and lacks large openings to enable access of the cytoskeleton to the remaining processes of silicification. Most centric species have substantially different silica morphologies and higher order assemblies on the distal valve surface compared with the base layer [2]. Since post-base layer processes may have less involvement by the cytoskeleton than in formation of the base layer, what cellular processes or components might be responsible for structural organization at this level? A physical factor could be expansion of the SDV in the z-axis distal direction, which will not only affect possible spatial constraints, but could alter the local chemical environment. A well-documented second factor is the involvement of silaffins, LCPAs, and silacidins in the process of silica formation. In some cases, silica precipitated *in vivo* by these organic molecules resembles the nanostructure of silica found in the distal surface of diatom valves, and models have been presented suggesting how meso- and nano-scale patterning could occur via the intermediary of these components [44]; [45]; [14]. In these experiments and models, no anchoring of the organics was done or postulated, thus many post-base layer processes could result from the types of chemical and electrostatic interactions that have been proposed for these molecules [44]; [45]; [14].

Precipitations of silica by silaffins and LCPAs *in vivo* have not resulted in formation of a higher-order regular assembly that closely resembles diatom mesoscale silica structures [10]; [13]; [11]; [12]. The correlation between actin filaments and mesoscale structures in a diverse variety of diatom species (Fig. 5, 9, 10, 13) indicates that actin is the major determinant of this. Since actin is located outside of the SDV, and silica polymerization determinants are located inside the SDV, there must be a means to interface between the two. Models have been proposed whereby actin or microtubules could affect processes internal to the SDV via the intermediary of proteins that interact with these cytoskeletal elements yet span the silicalemma and position polymerization determinants within the SDV [46]–[48]; [9]. The determinants could either provide a direct template for assembly or precipitation, or confine a space for precipitation [46]–[48]; [9]; [37]. Such models also explain why development of the valve occurs mostly in the z-axis distal direction because the initial polymerization determinants and deposited silica will be anchored on the proximal side by the actin and/or microtubule network. These networks may indeed be what are visibly associated with the mother cell valves in Figs. 2 and 5.

### Conclusions

Our results demonstrate a predominant role for the cytoskeleton in patterning meso- and micro-scale silica morphogenesis in diatoms. Actin microfilaments appear to be involved in the structuration of the silica both at the meso and the microscale and defining the limits of the SDV, while microtubules seem to be responsible for the microscale positioning of silica structures. One basic concept in materials science relates to the primary level of control over structure formation, the so called bottom up or top down approach. The bottom up approach uses assembly properties of small molecules or particles to form larger-scale structure. The top down approach uses an initial macro-scale structure which is then processed to generate smaller details. Previous work in diatoms characterizing the organic material closely associated with the silica highlighted their role in the “bottom up” processes of silica polymerization and nanoscale assembly [10]; [13]; [11]; [12]; [14]. The data presented here, along with previous work [6], indicates a substantial “top down” component of control over higher order structure formation that involves positioning within or molding of the SDV by the cytoskeleton. This is especially evident at the mesoscale, where the most distinct differences comparing silica structure in different diatom species occurs. Diatoms can make silica structures with a complexity that far exceeds current synthetic material approaches. Given the highly interactive nature of biological components and the propensity of biology to optimize processes, we think it likely that top down and bottom up processes in diatoms are highly integrated and that this contributes substantially to their capabilities to make such a wide variety of silica structures with precision on the nano- to micro-scales.

The insight into the role of cytoskeleton has important implications for the understanding of silica biomineralization. Additionally the finding that actin assembly in diatoms forms such a large variety of structures opens an interesting perspective regarding the study of actin dynamics and membrane interaction.

## Materials and Methods

### Diatom species and strains

*Coscinodiscus granii* CCMP1817, *Triceratium dubium*, *Entomoneis alata* CCMP1522, *Nitzschia curvilineata* CCMP555, and *Surirella* sp. CCMP2912 were obtained from the Provasoli-Guillard National Center for Culture of Marine Phytoplankton, Bigelow Laboratory for Ocean Sciences (West Boothbay Harbor, ME, USA), and maintained in NEPC medium (http://www.botany.ubc.ca/cccm/NEPCC/esaw.html). Stock cultures (50 mL) were maintained at 16°C–18°C on a 12∶12 light∶dark (L∶D) cycle. All other growth was in continuous light at an intensity of 150 µmol photons · m^2^ · s^−1^ at the same temperature. Synchronization of the different culture was performed as previously described (Hildebrand et al 2007). Briefly, cells were grown in silica free medium for 24 hrs, 400 µM of sodium silicate was added to the culture, and cell wall formation processes followed thereafter.

### Sample preparation for SEM and AFM

Diatom frustules were cleaned by acid treatment. Ten milliliters of diatom culture was harvested by centrifugation at 3,000×g for 4 min, rinsed once with 2.3% NaCl and frozen at −20°C. Acid treatment of cell walls was done by boiling cells in 1 ml concentrated sulfuric acid for 10 min, cooling, and then adding 20 mg KNO_3_, then boiling an additional 10 min. Frustules were then washed using centrifugation three times with ultra pure water. For SEM examination, samples were sputter coated with gold/palladium and observed with an FEI Quanta 600 (FEI Company, Hillsboro, OR, USA) scanning electron microscope at the Scripps Institution of Oceanography Unified Laboratory Facility. For AFM imaging, frustules were mounted on poly-L-lysine coated slides and imaged in air. Images were acquired in AC mode using a silicon cantilever with a spring constant of 42 N/m (AC160TS, Olympus) on a Veeco Bioscope catalyst Atomic Force Microscope coupled with a Zeiss inverted fluorescent microscope. AFM images were processed using WSxM 4.0 software [49].

### Sample preparation for fluorescence microscopy

Silica incorporation was visualized by addition in the culture medium of 100 ng · mL^−1^ of PDMPO ([2-(4-pyridyl)-5-((4-(2-dimethylaminoethylamino- carbamoyl)methoxy)phenyl)oxazole] (Invitrogen Corp., La Jolla, CA) prior to silicate replenishment [50]. Actin staining followed the procedure of van de Meene and Pickett-Heaps [25], where cells were fixed in 2% formaldehyde prepared in Actin Stabilizing Buffer (ASB – 10 mM PIPES/10 mM EGTA/5 mM MgSO_4_/pH 6.9) and containing 100 µM MBS (m-maleimidobenzoyl N- hydroxysuccinimide ester) and 1% NaCl at 4°C, rinsed twice in ASB buffer and stained with rhodamine phalloidin (Invitrogen Corp., La Jolla, CA) diluted at 1/100 in ASB buffer overnight at 4°C. Samples were then rinsed twice in ASB prior to observation. Microtubules were stained using antibodies. Cells were fixed in 0.5% glutaraldehyde in PBS buffer containing 1% NaCl at room temperature, then rinsed three times with the same buffer and incubated 1 hr in the same buffer containing 0.1% Triton X-100. Cells were then washed once and incubated 30 min in salted PBS buffer containing 1% BSA. Anti sea urchin α-tubulin from mouse (Santa Cruz Biotechnology, Santa Cruz CA, USA) was used at a dilution of 1/50 overnight at room temperature. Cells were rinsed three times in PBS buffer and incubated overnight at RT with the secondary antibodies (goat anti mouse labeled with Alexa fluor 546, Invitrogen Corp. Carlsbad CA, USA) diluted to 1/50 in 1% BSA containing buffer. Cells were rinsed three times prior to examination. Cells were imaged using a Zeiss AxioObserver inverted microscope equipped with an Apotome (Carl Zeiss Microimaging, Inc., Thornwood, NY, USA). The filter set used for PDMPO was Zeiss #21HE (Ex 387/15 nm, FT 409, Em 510/90 nm) and for rhodamine phalloidin, Zeiss #43HE (Ex 550/25 nm, FT 570 nm, Em 605/70 nm), respectively. With these filters and under the exposures used, no chlorophyll autofluorescence was visible. Chlorophyll was imaged using Zeiss filter set #16 (Ex 485/20 nm, FT 510 nm, Em 515 nm LP). Images were acquired with 40x/0.75 or 63x/1.4 objective oil immersion plan APO and treated using Axiovision 4.7.2 and Photoshop 5.0 software. Presented images are from 3D reconstructions (except when specified).

## References

[1] Norten TA, Melkonian M, Andersen RA (1996). Algal biodiversity.. Phycologia.

[2] Round FE, Crawford RM, Mann DG (1990). The diatoms: Biology and morphology of the genera..

[3] Poulsen NC, Spector I, Spurck TP, Shultz TF, Wetherbee R (1999). Diatoms gliding is the result of an actin-myosin motility system.. Cell Motil Cytoskeleton.

[4] Higgins MJ, Molino P, Mulvaney P, Wetherbee R (2003). The structure and nanomechanical properties of the adhesive mucilage that mediates diatom-substratum adhesion and motility.. J Phycol.

[5] Drum RW, Pankratz HS (1964). Post mitotic fine structure of *Gomphonema parvulum.*. J Ultrastruc Res.

[6] Pickett-Heaps J, Schmid A-MM, Edgar LA, Round FE, Chapman DJ (1990). The cell biology of diatom valve formation.. Progress in Phycological Research.

[7] Del Amo Y, Brzezinski MA (1999). The chemical form of dissolved Si taken up by marine diatoms.. J Phycol.

[8] Hildebrand M, York E, Kelz JI, Davis AK, Frigeri LG (2006). Nanoscale controle of silica morphology and three-dimensional structure during diatom cell wall formation.. J Mater res.

[9] Hildebrand M (2008). Diatoms, biomineralization processes, and genomics.. Chem Rev.

[10] Kröger N, Deutzmann R, Sumper M (1999). Polycationic peptides from diatom biosilica that direct silica nanosphere formation.. Science.

[11] Kröger N, Lorenz S, Brunner E, Sumper M (2002). Self-assembly of highly phosphorylated silaffins and their function in biosilica morphogenesis.. Science.

[12] Poulsen N, Kröger N (2004). Silica morphogenesis by alternative processing of silaffins in the diatom *Thalassiosira pseudonana.*. J Biol Chem.

[13] Kröger N, Deutzmann R, Bergsdorf C, Sumper M (2000). Species-specific polyamines from diatoms control silica morphology.. Proc Nat'l Acad Sci, USA.

[14] Wenzl S, Hett R, Richthammer P, Sumper M (2008). Silacidins: Highly acidic phosphopeptides from diatom shells assist in silica precipitation in vitro.. Angew Chem.

[15] Kröger N, Poulsen N (2008). Diatoms-From cell wall biogenesis to nanotechnology.. Annu Rev Genet.

[16] Eike B, Patrick R, Hermann E, Silvia P, Paul S (2009). Chitin-Based Organic Networks: An Integral Part of Cell Wall Biosilica in the Diatom *Thalassiosira pseudonana*.. Ang Chem Int ed.

[17] Pickett-Heaps JD, Tippit DH, Andreozzi JA (1979). Cell division in the pennate diatom *Pinullaria* IV. Valve morphogenesis.. Biol Cell.

[18] Schmid A-MM (1980). Valve morphogenesis in diatoms: a pattern-related filamentous system in pennates and the effect of APM, colchicine, and osmotic pressure.. Nova Hedwiga.

[19] Blank GS, Sullivan CW (1983). Diatom mineralization of silicic acid VI. The effects of microtubule inhibitors on silicic acid metabolism in *Navicula saprophila.*. J Phycol.

[20] Cohn SA, Nash J, Pickett-Heaps JD (1989). The effect of drugs on diatom valve morphogenesis.. Protoplasma.

[21] Pickett-Heaps JD, Cohn SA, Schmid A-M, Tippit DH (1988). Valve morphogenesis in *Surirella* (Bacillariophyceae).. J Phycol.

[22] Pickett-Heaps JD, Wetherbee R, Hill DRA (1988). Cell division and morphogenesis of the labiate process in the centric diatom *Ditylum brightwellii*.. Protoplasma.

[23] Pickett-Heaps JD, Carpenter J, Koutoulis A (1994). Valve and setae (spine) morphogenesis in the centric diatom *Chaetoceros peruvianus* Brightwell.. Protoplasma.

[24] Pickett-Heaps JD (1998). Cell division and morphogenesis of the centric diatom *Chaetoceros decipiens* (Bacillariophyceae). II. Electron microscopy and a new paradigm for tip growth.. J Phycol.

[25] van de Meene AML, Pickett-Heaps JD (2002). Valve morphogenesis in the centric diatom *Proboscia alata* Sundstrom.. J Phycol.

[26] Van de Meene AML, Pickett-Heaps JD (2004). Valve morphogenesis in the centric diatom *Rhizosolenia setigera* (Bacillariophyceae, Centrales) and its taxonomic implications.. Eur J Phycol.

[27] Tesson B, Hildebrand M (2010). Dynamics of silica cell wall morphogenesis in the diatom *Cyclotella cryptica*: Substructure formation and the role of microfilaments.. J Struct Biol.

[28] Walsh CJ (2007). The role of actin, actomyosin and microtubules in defining cell shape during the differentiation of *Naegleria amebae* into flagellates.. Eur J Cell Biol.

[29] Fletcher DA, Mullins D (2010). Cell mechanics and the cytoskeleton.. Nature.

[30] Schmid A-M, Volcani BE (1983). Wall morphogenesis in *Coscinodiscus wailesii* Gran and Angst. I. Valve morphology and development of its architecture.. J Phycol.

[31] Losic D, Rosengarten G, Mitchell JG, Voelcker NH (2006). Pore architecture of diatom frustules: Potential nanostructured membranes for molecular and particle separations.. J Nanosci Nanotech.

[32] Losic D, Short K, Mitchell JG, Lal R, Voelcker NH (2007). AFM nanoindentations of diatom biosilica surfaces.. Langmuir.

[33] Pollock F, Pickett-Heaps J (2005). Spatial determinants in morphogenesis: Recovery from plasmolysis in the diatom *Ditylum*.. Cell Motil Cytoskeleton.

[34] Pickett-Heaps JD, Kowalski SE (1981). Valve morphogensis and the microtubule center of the diatom *Hantzschia amphioxysis*.. Eur J Cell Biol.

[35] Pickett-Heaps JD (1983). Valve morphogenesis and the microtubule center in three species of the diatom *Nitzschia*.. J Phycol.

[36] Mann DG, Mann DG (1984). An ontogenetic approach to diatom systematics.. Proceedings of the seventh international diatom symposium.

[37] Hildebrand M, Kim S, Shi D, Scott K, Subramaniam S (2009). 3D imaging of diatoms with ion-abrasion scanning electron microscopy.. J Struct Biol 166.

[38] Schmid A-M, Schulz D (1979). Wall morphogenesis in diatoms: deposition of silica by cytoplasmic vesicles.. Protoplasma.

[39] Pickett-Heaps J (1998). Cell division and morphogenesis of the centric diatom *Chaetoceros decipiens* (Bacillariophyceae) II. Electron microscopy and a new paradigm for tip growth.. J Phyco.

[40] Revenu C, Athman R, Robine S, Louvard D (2004). The co-workers of actin filaments: From cell structures to signals.. Nat Rev Mol Cell Biol.

[41] Saarikangas J, Zhao HX, Lappalainen P (2010). Regulation of the actin cytoskeleton-plasma membrane interplay by phosphoinositides.. Physiol Rev.

[42] Lanzetti L (2007). Actin in membrane trafficking.. Current opinion in cell biology.

[43] McMahon HT, Gallop JL (2005). Membrane curvature and mechanisms of dynamic cell membrane remodelling.. Nature.

[44] Kröger N, Sumper M (1998). Diatom cell wall proteins and the cell biology of silica biomineralization.. Protist.

[45] Sumper M (2002). A phase separation model for the nanopatterning of diatom biosilica.. Science.

[46] Robinson DH, Sullivan CW (1987). How do diatoms make silicon biominerals?. Trends in Biochemical Sciences.

[47] Hildebrand M, Frigeri LG, Davis AK (2007). Synchronized growth of *Thalassiosira pseudonana* (Bacillariophyceae) provides novel insights into cell-wall synthesis processes in relation to the cell cycle.. J Phycol.

[48] Davis AK, Hildebrand M, Sigel HS, A (2008). Molecular processes of biosilicification in Diatoms.. Metal Ions in Life Sciences, Volume 4 From Nature to Application.

[49] Horcas I, Fernandez R, Gomez-Rodriguez JM, Colchero J, Gomez-Herrero J (2007). WSXM: A software for scanning probe microscopy and a tool for nanotechnology.. Review of Scientific Instruments.

[50] Shimizu K, Del Amo Y, Brzezinski MA, Stucky GD, Morse DE (2001). A novel fluorescent silica tracer for biological silicification studies.. Chem Biol.

